# Diagnostic and Therapeutic Approach in a Metastatic Vaginal Adenocarcinoma: A Case Report

**DOI:** 10.3389/fimmu.2021.686879

**Published:** 2021-07-23

**Authors:** Eva Katharina Egger, Damian J. Ralser, Kira Lindner, Florian Recker, Milka Marinova, Oleksandre Savchenko, Jan-Frederic Lau, Alexander Mustea

**Affiliations:** ^1^ Department of Gynecology and Gynecological Oncology, University Hospital Bonn, Bonn, Germany; ^2^ Department of Diagnostic and Interventional Radiology, University Hospital Bonn, Bonn, Germany; ^3^ Department of Pathology, University Hospital Bonn, Bonn, Germany

**Keywords:** immune checkpoint blockade, vaginal adenocarcinoma, trastuzumab, abscopal effect, mutational burden

## Abstract

**Background:**

Vaginal adenocarcinomas (VAC) are most often reported after intrauterine exposition to diethylstilbestrol (DES). Rarely, VACs are reported as a malignant transformation of vaginal adenosis or endometriosis, in the context of chromosomal abnormalities or malformations of the uterus or the vagina. VACs without DES exposition have a poor prognosis and a significantly worse outcome compared to vaginal squamous cell carcinomas or DES-associated VACs.

**Objective:**

Here, we report the case of a primarily metastatic VAC, treated successfully with different lines of chemo-, antiangiogenic, antibody, and immunotherapy.

**Case:**

The 49-year-old patient presented in 5/2018 with a primarily pulmonary metastatic VAC. Significant tumor reduction was seen after six cycles of carboplatin AUC5/paclitaxel 175 mg/m²/bevacizumab 15 mg/kg q3w. Bevacizumab maintenance therapy and later cisplatin mono 50 mg/m² q2w led to local and distant tumor progression. To identify a potential targeted therapy, new tumor biopsies were obtained. Immunohistochemistry revealed ERBB2 expression, and paclitaxel 80 mg/m² weekly plus trastuzumab 4 mg/m² respectively 2 mg/m² q3w was administered. Due to local and pulmonal tumor progression after 6 months and persistent ERBB2 positivity, the therapy was adjusted to trastuzumab emtansine (T-DM1) 3.6 mg/kg q3w; however, the patient remained locally progressive after three cycles of T-DM1 and additionally showed a new bone metastasis. The new tumor biopsies revealed a combined positive score (CPS) of 2 regarding PD-L1, and pembrolizumab 200 mg q3w was initiated. The bone metastasis was radiated and treated with denosumab 120 mg q4w. Extreme tumor regression followed by stable disease was maintained for 9 months. Due to a slow locoregional progress only with new inguinal lymph node and pararectal lymph node metastases, a new tumor biopsy was taken. Molecular profiling showed an ARID1A mutation, a mutational burden of 5.1 mutations per megabase, and no genfusions. Based on these findings, therapy with PD-L1 antibodies, PD-1 antibodies, gemcitabine, or dasatinib was suggested. Therefore, administration of pembrolizumab was continued and local radiation therapy was performed. This led to a decrease in local tumor manifestations and a stable systemic disease.

**Conclusion:**

Our case demonstrates the diagnostic and therapeutic approach in a patient with primary metastatic vaginal adenocarcinoma. By tumorgenetic profiling, different lines of systemic therapy, namely, antiangiogenic therapy, monoclonal antibody therapy, immunotherapy, and local radiation therapy, were identified and successfully administered.

## Background

Less than 0.1% of all malignancies in women are accounting for primary carcinomas of the vagina (1). The majority of all vaginal carcinomas are squamous cell carcinomas. Vaginal adenocarcinomas (VAC) are most often reported after intrauterine exposition to diethylstilbestrol (DES) (2), rarely in the context of vaginal adenosis (3, 4) or in the context of chromosomal abnormalities or malformations of the uterus or the vagina (5). The largest study on VACs without DES exposition showed a poor prognosis with significantly worse survival compared to squamous cell carcinomas or DES-associated VACs. Histologically, clear cell, mucinous, endometrioid, and no further specified subtypes are distinguished (6). Data regarding risk factors as endocrine disruptors or enhanced inflammation are missing for this disease (7). Due to the rarity of the disease, there are no data on therapeutic options in the metastatic setting.

Here, we report the case of a primarily metastatic VAC without any history of DES, adenosis, or chromosomal abnormalities. Based on repeated immunohistochemistry and tumorgenetic profiling, different lines of chemo-, antiangiogenic, antibody, and immunotherapy were identified and successfully administered for almost 3 years by now.

## Case Report

**Figure 1:**
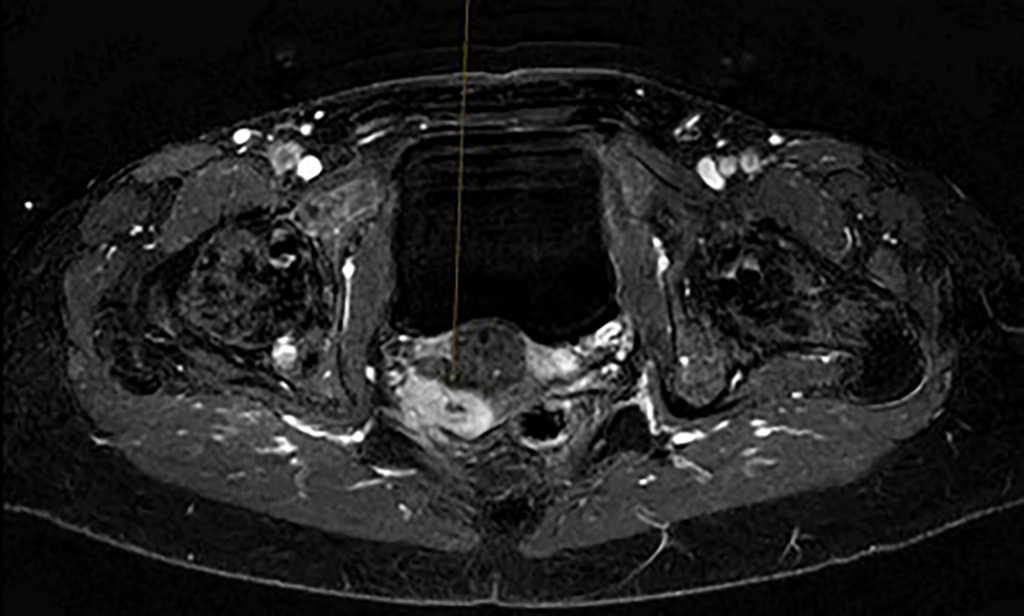
Initial magnet resonance Imaging scan of the pelvis.

**Figure 2:**
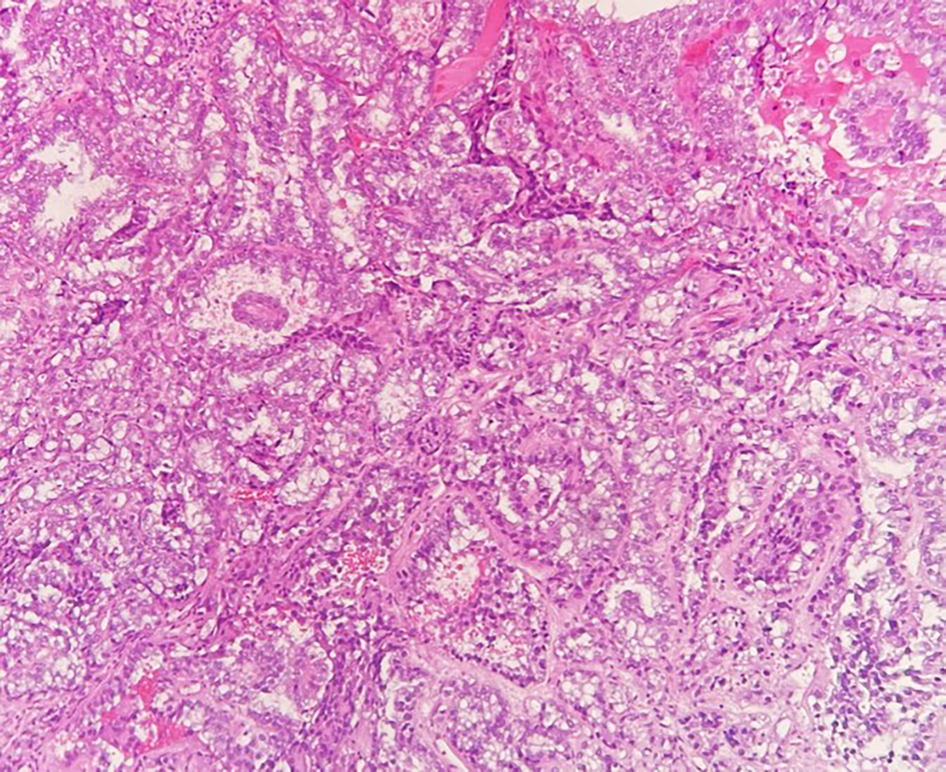
Hematoxilyn and eosin stained clear cell adenocarcinoma biopsy.

A 49-year-old patient was diagnosed with a 4 cm clear cell adenocarcinoma of the vagina (VAC; for patient characteristics see **Table 1**) with tumor infiltration of the lateral pelvic side wall (see **Figures 1**, **2**). At the time of primary diagnosis, multiple lung metastases of up to 5 mm in size were detected in a computer tomography (CT) scan. A CT-guided puncture of the lung metastases did not appear feasible due to the small size of the metastatic lesions. Immunohistochemistry of the primary tumor showed negative hormone-receptor status, negative p16 expression, and a high Ki-67 of 70%. Due to the locally advanced, inoperable VAC with shooting pain into the right leg and the metastatic setting, systemic therapy with carboplatin AUC5/paclitaxel 80 mg/m², and bevacizumab 15 mg/kg q3w was induced. This resulted in clinical and image-morphological tumor regression. After completion of chemotherapy for six cycles, bevacizumab 15 mg/kg maintenance therapy was continued. Clinical examination and imaging revealed local and pulmonal disease progression after four cycles of bevacizumab 15 mg/kg alone. Repeated tumor biopsy for further immunohistochemistry showed all mismatch repair proteins stable, a PD-L1 combined positive score (CPS) of 2, and an ERBB2 expression.

**Table 1:** Medical history and clinical, histological, and molecular characteristics of the patient.

History of primary diagnosis and medical history	
Gender, age	Female, 49 years
Staging of primary status	Primary pulmonary metastatic VAC
	Tumor diameter: 4 cm with infiltration of the lateral pelvic side wall due to an additional tumor on the sacrotuberal ligament
	Multiple lung metastases up to 5 mm diameter
Immunohistochemistry	Vimentin +8
	CK 7 +
	PR−, ER−
	p16−
	Ki-67: 70%
	ERBB2: Expression 60%
	PD-L1: CPS: 2, IC: 5, TPS: <1%
	MLH1, MSH2, MSH6, PMS2 stable
Molecular profile	ARID1A Exon 20: c.5548dup (p.D1850fs) 5,1Mut/Mb
	No genefusions
Performance Status	ECOG 0
Medical history	None, especially no history of DES exposure, endometriosis, or HPV infection
Family history	None
Psychological history	Married, 1 child

Therefore, treatment with cisplatin mono 50 mg/kg q2w was initiated. After only two cycles of cisplatin, the local progress was unmistakable. Imaging showed a new osteolytic metastasis in the right-sided ascending branches of the ischial bone, a new 6 mm liver metastasis in the segment 8, and a new left-sided supraclavicular lymph node metastasis, in addition to local and pulmonal progression.

In agreement with the patient, trastuzumab 4 mg/kg initially, followed by 2 mg/kg q3w and paclitaxel 80 mg/m² weekly, was started, as well as denosumab 120 mg q4w subcutaneously. After three cycles, clinical evaluation revealed a good response, which showed concordance in imaging except for a progression of the osteolytic metastasis. Therefore, intensity modulated radiotherapy with 30 Gy was additionally initiated. After 6 months of therapy, local and distant tumor progression was observed again. As the repeated tumor biopsy still revealed ERBB2 expression, trastuzumab emsantine (T-DM1) 3.6 mg/kg q3w was administered for three cycles but unfortunately without success.

Due to the CPS of 2, therapy was adjusted to pembrolizumab 200 mg q3w. After only 4 months of therapy, pulmonal and lymph node metastases became invisible, the vaginal tumor became almost invisible, and the patient remained completely stable for 9 months. Subsequently, the patient developed a slow locoregional progress with new inguinal and pararectal lymph node metastases, while systemic disease remained stable. A new biopsy was obtained for further molecular genetic profiling of the tumor (Molecular Health Guide). The analysis showed an ARID1A mutation in exon 20: c.5548dup. (p.D1850fs), a mutational burden of 5.1 Mut/Mb, and no gene fusions. Based on these findings, PD-L1 antibodies, PD-1 antibodies, gemcitabine, or dasatinib was the recommended therapeutic option. As distant disease had remained stable, local progress was comparably slow, and the patient refused chemotherapeutic options as gemcitabine, pembrolizumab 200 mg q3w was continued and local radiation therapy with 5 × 5Gy was initiated. Radiation therapy achieved a considerable regression of the local progress, while distant disease remained stable with prolonged pembrolizumab 200 mg q3w with still ongoing response.

According to iRECIST (Response Evaluation Criteria in Solid Tumors in Immunotherapy), the actual timepoint response is considered as “iPartial Response”. The tumor load of the Target lesion is reduced by −47.7% compared to the nadir. At the moment there is no new lesion. According to iRECIST the target lesion in the vagina has decreased after the addition of radiation therapy by −32.9%.

**Figure 3:**
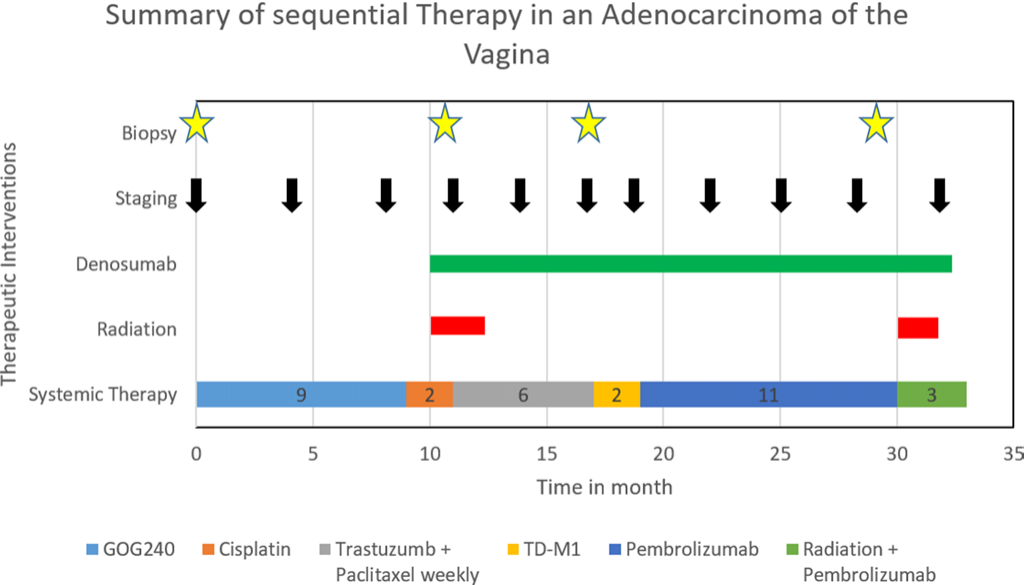
Summary of sequential therapy in an adenocarcinoma of the vagina.

**Figure 3** shows all sequential therapies.

## Discussion

Vaginal adenocarcinomas are extremely rare, and their incidence has declined since diethylstilbestrol has been identified as a promoter of this disease (2, 6). Further suspected disease promoters are a vaginal adenosis and endometriosis. Nevertheless, gastric-type mucinous adenocarcinomas have also been described (6). While DES-related VACs show an overall survival of 78%, non-DES-related VACs have a poor prognosis with 5-year overall survival rates of 35% (2, 6, 8). Furthermore, distant metastases are more frequently observed in VAC than in vaginal squamous cell carcinoma, preferably in the lung, the bones, or the supraclavicular lymph nodes (6).

The SEER analysis showed no response improvement whenever radiotherapy was expanded by chemotherapy (9). Therefore, therapeutic recommendations in our case have been rather scarce.

The Moore criteria (pelvic disease, prior radiation therapy, age, race, and ECOG above 1) identified patients with cervical cancer that might least respond to systemic therapy as chemotherapy and antiangiogenic therapy. Likewise, if only one risk factor was present, patients had a favorable outcome (10). As the 4 cm pelvic tumor was the only criterion our patient showed, we therefore opted to treat her analogously to cervical cancer with the GOG 240 protocol paclitaxel 175 mg/m²/carboplatin AUC5/Bevacizumab 15 mg/kg q3w. Cisplatin was replaced by carboplatin due to less toxicity (11, 12). As bevacizumab only failed to stabilize the disease, cisplatin mono 50 mg/m² q2w i.v. was administered, which is typically used in the palliative setting of cervical cancer (12).

ERBB2 mutations are a frequent event in solid tumors, and the combination of trastuzumab and lapatinib has shown promising antitumoral effects in four different ERBB2-positive tumor entities as adenosarcoma, endometrial cancer, cholangiocarcinoma, and colorectal cancer (13). Research has also shown ERBB2 expression in about 5–21% of cervical cancer specimen, and treatment with lapatinib and trastuzumab inhibited tumor growth significantly in a patient-derived xenograft model for cervical cancer. Thus, we decided to screen for ERBB2 expression in our patient (14–16). Trastuzumab 4 mg/kg initially, followed by 2 mg/kg q3w and paclitaxel 80 mg/m² weekly, kept the disease stable for 6 months. In the case of further progression, therapy was adjusted to trastuzumab emsantine (T-DM1) 3.6 mg/kg q3w, analogous to the second-line treatment in ERBB2-overexpressing metastatic breast cancer (17). Treatment with T-DM1 showed no reduction in tumor size. Despite persistent ERBB2 expression, we decided against the abovementioned combination of trastuzumab and lapatinib.

Due to a CPS of 2 regarding PD-L1, immunotherapy with pembrolizumab was initiated. The rationale for this therapeutic decision was the Keynote 158 study (18), which showed an overall response rate of 12.2% to pembrolizumab in cervical cancer patients with a CPS above 1. In that study, the median duration of response was not reached (18). The mutational burden analysis of the entire Keynote 158 cohort showed a significant difference in objective response of 29% in the high mutational burden group (more than 10 mutations per megabase) and 6% in the low mutational burden group (19). Although our patient showed a mutational burden of only 5.1 Mut/Mb, she responded well. Vaginal carcinomas were not included in the Keynote 158 study. The Checkmate 358 trial included five patients with vulvar/vaginal cancer, but all had squamous cell histology and only one out of five patients responded for only 5 months (20). As our patient progressed locally in the further course, we hoped to overcome local resistance by local radiation therapy using the abscopal effect. It is presumed that irradiation leads to cell death by liberating tumor antigens of irradiated tumor cells, which might activate antigen-presenting cells and cytotoxic T-cells expressing PD-1 on their surface. Therefore, it is presumed that the therapeutic effects of radiation therapy and immunotherapy may be enhanced by the combination of both (21). In our case, the patient showed in clinical evaluation and imaging a distinct reduction of the vaginal tumor and the inguinal lymph node metastasis while distant disease remained stable.

We consider our patient as longtime survivor in a metastatic setting with long-term immunotherapy. Therefore, the question arises how much supportive care regarding ascorbic acid and resveratrol, for example, may be useful to prevent inflammatory processes and enhance the therapeutic effect in the further course (22, 23).

Taken together, we demonstrate the diagnostic and therapeutic approach in a rare case of a primary metastatic VAC. Administration of different lines of chemotherapy, antiangiogenic therapy, monoclonal antibody therapy, immunotherapy, and local radiation therapy has an ongoing, long-lasting effect on overall survival in this patient. Generally, our case demonstrates the extent to which targeted tumor genetic characterization can positively influence the therapy and course of rare tumor entities.

## Data Availability Statement

The original contributions presented in the study are included in the article/supplementary material. Further inquiries can be directed to the corresponding author.

## Ethics Statement

Written informed consent was obtained from the individual(s) for the publication of any potentially identifiable images or data included in this article.

## Author Contributions

EE and AM were responsible for study design and concept. DR, KL, FR, OS, and MM were involved in data acquisition. AM revised the manuscript. All authors contributed to the article and approved the submitted version.

## Conflict of Interest

The authors declare that the research was conducted in the absence of any commercial or financial relationships that could be construed as a potential conflict of interest.

## Publisher’s Note

All claims expressed in this article are solely those of the authors and do not necessarily represent those of their affiliated organizations, or those of the publisher, the editors and the reviewers. Any product that may be evaluated in this article, or claim that may be made by its manufacturer, is not guaranteed or endorsed by the publisher.
